# Modeling is Believing? How AlphaFold2 Can Mislead Molecular Interpretation

**DOI:** 10.1063/4.0001049

**Published:** 2025-10-27

**Authors:** Karly Forker, Matthew C Fleming, Kenneth H Pearce, Cyrus Vaziri, Albert Bowers, Pei Zhou

**Affiliations:** 1 Duke University, Durham, NC, USA; 2 University of North Carolina Chapel Hill, Chapel Hill, NC, USA

## Abstract

The germ cell protein MAGEA4 is mis-expressed in many cancer cells, where it binds and stabilizes RAD18, an E3 ubiquitin ligase essential for the translesion synthesis (TLS) DNA repair pathway. Cancer cells’ dependence on the MAGEA4-RAD18 interaction makes it an attractive target for structural investigation to aid in therapeutic development. Previous work by Griffith-Jones et al. used NMR titration and AlphaFold2 modeling to propose that a short peptide of RAD18 interacts with MAGEA4 in a specific orientation. In contrast, our crystal structure of the complex revealed a completely flipped binding orientation. Utilizing AlphaFold-Multimer, we generated additional models, identifying two binding clusters: one consistent with Griffith-Jones' model and the other still producing a flipped helix. Despite the differing orientations, key interfacial residues overlap. This study offers an important example of the limitations of AlphaFold2, especially with short, hydrophobic peptides and highlights the importance of experimental validation for computational models. Our findings clarify the MAGEA4-RAD18 interaction, aiding in the development of inhibitors targeting this complex for cancer therapy.

